# Cutaneous T-cell-attracting chemokine as a novel biomarker for predicting prognosis of idiopathic pulmonary fibrosis: a prospective observational study

**DOI:** 10.1186/s12931-021-01779-9

**Published:** 2021-06-17

**Authors:** Takafumi Niwamoto, Tomohiro Handa, Yuko Murase, Yoshinari Nakatsuka, Kiminobu Tanizawa, Yoshio Taguchi, Hiromi Tomioka, Keisuke Tomii, Hideo Kita, Michihiro Uyama, Michiko Tsuchiya, Masahito Emura, Tetsuji Kawamura, Naoki Arai, Machiko Arita, Kazuko Uno, Akihiko Yoshizawa, Ryuji Uozumi, Izumi Yamaguchi, Fumihiko Matsuda, Kazuo Chin, Toyohiro Hirai

**Affiliations:** 1 Department of Respiratory Medicine, Kyoto University Graduate School of Medicine, 54 Shogoin Kawahara-cho, Sakyo-ku, Kyoto, 606-8507 Japan; 2 Department of Advanced Medicine for Respiratory Failure, Kyoto University Graduate School of Medicine, 54 Shogoin Kawahara-cho, Sakyo-ku, Kyoto, 606-8507 Japan; 3 Department of Respiratory Care and Sleep Medicine, Kyoto University Graduate School of Medicine, 54 Shogoin Kawahara-cho, Sakyo-ku, Kyoto, 606-8507 Japan; 4 Department of Respiratory Medicine, Tenri Hospital, 200 Mishima-cho, Nara, 632-0015 Japan; 5 Department of Respiratory Medicine, Kobe City Medical Center West Hospital, 2-4 Ichiban-cho, Nagata-ku, Hyogo, 653-0013 Japan; 6 Department of Respiratory Medicine, Kobe City Medical Center General Hospital, 2-1-1 Minatojimaminami-machi, Chuou-ku, Hyogo, 650-0047 Japan; 7 Department of Respiratory Medicine, Takatsuki Red Cross Hospital, 1-1-1 abuno, Osaka, 569-1045 Japan; 8 Respiratory Disease Center, Kitano Hospital, Tazuke Kofukai Medical, Research Institute, 2-4-0 Ohgimachi, Kita-ku, Osaka, 530-8480 Japan; 9 Department of Respiratory Medicine, Otowa Hospital, 2 Otowachinji-cho, Yamashina-ku, Kyoto, 607-8062 Japan; 10 Department of Respiratory Medicine, Kyoto City Hospital, 1-2 Mibuhigasitakada-cho, nakagyo-ku, Kyoto, 604-8845 Japan; 11 Department of Respiratory Medicine, Himeji Medical Center, 68 Hon-machi, Hyogo, 670-8520 Japan; 12 National Hospital Organization Ibaraki Higashi National Hospital, Terunuma 825, Tokai, Ibaraki 319-1113 Japan; 13 Department of Respiratory Medicine, Ohara Healthcare Foundation, Kurashiki Central Hospital, 1-1-1 Miwa, Kurashiki, Okayama 710-8602 Japan; 14 Louis Pasteur Center for Medical Research, 103-5 Tanakamonzen-cho, Sakyo-ku, Kyoto, 606-8225 Japan; 15 Department of Diagnostic Pathology, Kyoto University Graduate School of Medicine, 54 Shogoin Kawahara-cho, Sakyo-ku, Kyoto, 606-8507 Japan; 16 Department of Biomedical Statistics and Bioinformatics, Kyoto University Graduate School of Medicine, 54 Shogoin Kawahara-cho, Sakyo-ku, Kyoto, 606-8507 Japan; 17 Center for Genomic Medicine, Kyoto University Graduate School of Medicine, 53 Shogoin Kawahara-cho, Sakyo-ku, Kyoto, 606-8507 Japan

**Keywords:** CTACK, Cutaneous T-cell-attracting chemokine, CCL27, IPF, Idiopathic pulmonary fibrosis, Biomarker, CC chemokine receptor 10, Chemokine, Cytokine, Multiplex

## Abstract

**Background:**

Idiopathic pulmonary fibrosis (IPF) is a chronic, progressive fibrotic lung disease that leads to respiratory failure and death. Although there is a greater understanding of the etiology of this disease, accurately predicting the disease course in individual patients is still not possible. This study aimed to evaluate serum cytokines/chemokines as potential biomarkers that can predict outcomes in IPF patients.

**Methods:**

A multi-institutional prospective two-stage discovery and validation design using two independent cohorts was adopted. For the discovery analysis, serum samples from 100 IPF patients and 32 healthy controls were examined using an unbiased, multiplex immunoassay of 48 cytokines/chemokines. The serum cytokine/chemokine values were compared between IPF patients and controls; the association between multiplex measurements and survival time was evaluated in IPF patients. In the validation analysis, the cytokines/chemokines identified in the discovery analysis were examined in serum samples from another 81 IPF patients to verify the ability of these cytokines/chemokines to predict survival. Immunohistochemical assessment of IPF-derived lung samples was also performed to determine where this novel biomarker is expressed.

**Results:**

In the discovery cohort, 18 cytokines/chemokines were significantly elevated in sera from IPF patients compared with those from controls. Interleukin-1 receptor alpha (IL-1Rα), interleukin-8 (IL-8), macrophage inflammatory protein 1 alpha (MIP-1α), and cutaneous T-cell-attracting chemokine (CTACK) were associated with survival: IL-1Rα, hazard ratio (HR) = 1.04 per 10 units, 95% confidence interval (95% CI) 1.01–1.07; IL-8, HR = 1.04, 95% CI 1.01–1.08; MIP-1α, HR = 1.19, 95% CI 1.00–1.36; and CTACK, HR = 1.12 per 100 units, 95% CI 1.02–1.21. A replication analysis was performed only for CTACK because others were previously reported to be potential biomarkers of interstitial lung diseases. In the validation cohort, CTACK was associated with survival: HR = 1.14 per 100 units, 95% CI 1.01–1.28. Immunohistochemistry revealed the expression of CTACK and CC chemokine receptor 10 (a ligand of CTACK) in airway and type II alveolar epithelial cells of IPF patients but not in those of controls.

**Conclusions:**

CTACK is a novel prognostic biomarker of IPF.

*Trial registration* None (because of no healthcare intervention)

**Supplementary Information:**

The online version contains supplementary material available at 10.1186/s12931-021-01779-9.

## Background

Idiopathic pulmonary fibrosis (IPF) is chronic, progressive interstitial pneumonia of unspecified cause with median survival of 2–3 years from diagnosis [1]. However, the clinical course varies by case. The search for biomarkers is an important topic in IPF research, and its significance includes disease classification and diagnosis, surrogate treatment endpoints, treatment efficacy estimation, and accurate outcome estimation. However, no blood biomarkers that serve as prognostic factors have been established. Blood biomarkers are advantageous since they are non-invasive, and data are reproducible. Moreover, biomarker studies may contribute to the understanding of the pathogenesis of IPF.

IPF is associated with repeated micro-injury of the alveolar epithelium and abnormal tissue repair, a process in which fibroblasts differentiate into myofibroblasts and over-secrete an extracellular matrix, which is believed to function in the pathogenesis of IPF [2]. Regarding blood biomarkers related to the disease process, previous studies have reported that baseline or longitudinal changes in the expression of the following markers are associated with IPF prognosis: matrix metalloproteinases (MMPs), such as MMP-7 [3–5] and MMP-10 [6]; Krebs von den Lungen-6 (KL-6); surfactant protein-D (SP-D) produced by type II alveolar epithelial cells [7]; intercellular adhesion molecule-1 [3]; epithelial cell markers, such as carbohydrate antigen 19–9 [4] and carcinoembryonic antigen [8]; and periostin [9], a component of the extracellular matrix. Among the cytokines/chemokines, chemokine CC motif ligand 18 (CCL18) [10] and interleukin-8 (IL-8) [3] have been reported to be associated with IPF prognosis, but comprehensive evaluation of cytokines/chemokines as biomarkers of IPF has not been performed.

Therefore, we aimed to identify biomarkers to predict IPF prognosis. We comprehensively measured 48 cytokines and chemokines using a multiplex assay in sera from IPF patients and investigated their association with disease severity and survival.

## Methods

### Study subjects

The discovery cohort comprised 100 IPF patients who were prospectively enrolled at Kyoto University Hospital between February 2008 and January 2017. IPF was diagnosed by multidisciplinary consensus according to established guidelines [11]. Patients who were diagnosed previously were also reevaluated based on the new diagnostic criteria [11]. Patients were excluded if they had active neoplastic disease or an acute worsening, such as an acute exacerbation of IPF, infection, and congestive heart failure, at the time of enrollment, or when they had previously undergone therapeutic lung resection for malignant pulmonary disease. Patients were initially evaluated according to the modified Medical Research Council dyspnea scale (graded from 0 to 4); they also underwent standardized pulmonary function tests [12] and a 6-min walk test (6MWT) [13]. Arterial blood gas (ABG), complete blood count, biochemical measurements, serum biomarkers (KL-6 [14, 15], SP-D [7], and lactate dehydrogenase), and, if necessary, BALF, were also evaluated. The 6MWT was performed, and ABG was tested in normal room air. After these initial evaluations, patients visited the outpatient clinic every 3 to 6 months. Therapeutic decisions were made on an individual basis with no universal therapeutic protocol.

Blood samples were obtained at the time of initial evaluation. BAL was performed within 6 months from the initial evaluation in patients who experienced no acute exacerbation. Blood and BAL samples were centrifuged immediately following collection, and serum and BAL supernatants were stored at − 80 °C until further analysis. Serum samples were also obtained from 32 healthy volunteers (control group). The validation cohort comprised 81 treatment-naïve IPF patients who were prospectively enrolled at Kyoto University Hospital, Tenri Hospital, Kobe City Medical Center West Hospital, Kobe City Medical Center General Hospital, Takatsuki Red Cross Hospital, Kitano Hospital, Otowa Hospital, Kyoto City Hospital, and Himeji Medical Center between October 2013 and July 2019. Exclusion criteria, initial and follow-up evaluations, therapeutic decision-making, and blood sampling were the same as those in the discovery cohort. The validation and discovery cohorts were mutually exclusive.

This prospective registry study including discovery and validation cohorts was approved by the Institutional Review Board of Kyoto Universities (G1059) and other collaborative institutions, and all study participants provided written informed consent.

### Multiplex cytokine detection

Overall, 100 serum and 30 BALF samples from the discovery cohort and 32 serum samples from the control group were assayed for 48 cytokines using the Bio-Plex Suspension Array System with Bio-Plex Pro Human Cytokine Screening 48-Plex Panel (Bio-Rad Laboratories Inc., CA, USA). The assay tested for the following cytokines: IL-1β, IL-1Ra, IL-1α, IL-2, IL-2Rα, IL-3, IL-4, IL-5, IL-6, IL-7, IL-8, IL-9, IL-10, IL-12p40, IL-12p70, IL-13, IL-15, IL-16, IL-17, IL-18, Eotaxin, FGF basic, G-CSF, GM-CSF, IFN-γ, GRO-α, HGF, IFN-α2, LIF, MCP-3, IP-10, MCP-1, MIG, β-NGF, SCF, SCGF-β, SDF-1α, MIP-1α, MIP-1β, PDGF-BB, RANTES, TNF-α, VEGF, CTACK, MIF, TRAIL, M-CSF, and TNF-β. Cytokines were excluded from the analyses when more than 50% of values were outside the upper or lower limits of detection. If the value was less than the lowest detection value, 50% of the lowest detection value was applied [4].

Based on the results in the discovery cohort, CTACK was measured in sera from the validation cohort using the Bio-Plex Pro Human Chemokine CTACK/CCL27 kit (Bio-Rad Laboratories Inc., CA, USA).

### Visual assessment of combined pulmonary fibrosis and emphysema

The extent of emphysema was independently scored by two observers (THanda. and TN) who withheld clinical information, as described previously [16]. Briefly, emphysema was defined as a highly viscous lung region with no distinct walls. The emphysema score was calculated by visually estimating the percentage of emphysema in the upper, middle, and lower part of each lung by 10% and averaging them to calculate the total emphysema score. For the IPF patients in the discovery cohort, the visual assessment of combined pulmonary fibrosis and emphysema (CPFE) was diagnosed when the total emphysema score was ≥ 10%. Interobserver disagreement in the diagnosis of CPFE was resolved via consensus.

### Immunohistochemistry

To determine the cellular location of CTACK and CC chemokine receptor 10 (CCR10, a ligand of CTACK), immunohistochemistry for CTACK and CCR10 was performed. Lung tissue samples from five patients with IPF (stored at Kyoto University Hospital with patient consent) and healthy controls were analyzed by immunohistochemistry according to a routine method (supplement). Lung tissue samples from IPF patients were obtained during lung transplantation procedures performed at Kyoto University Hospital, whereas tissues from non-IPF controls were purchased from OriGene (MA, USA. catalog no. CB715157). Then, 5-μm-thick paraffin-embedded tissue sections were analyzed using the Avidin–biotin-peroxidase complex method. For antigen retrieval, the slides were immersed in a citrate buffer and heated in a microwave. A mouse anti-human chemokine CC motif ligand 27 (CCL27, also designated as CTACK) antibody (sc390112, Santa Cruz Biotechnology, CA, USA) and a goat anti-human CCR10 antibody (ab3944, Abcam, Cambridge, UK) were applied as primary antibodies (1:200 dilution). Positive staining was visualized using 3,3’-diaminobenzidine. A pulmonary pathologist (AY) evaluated the localization of CTACK and CCR10 expression in IPF lung tissues and compared the results observed in control specimens.

### Statistical analysis

Data of clinical features and multiplex measurements were reported as medians (interquartile ranges [IQRs]) or numbers (percentages), as appropriate. Cytokine values were compared between the discovery cohort and the control group through analysis of covariance to adjust for age, sex, and smoking history, and the results were corrected using the false discovery rate (FDR) method (Benjamini and Hochberg method) [17],which controls the proportion of false positives among the set of rejected hypothesis. In contrast to familywise error rate controlling procedures that guard against any false positives, FDR method provides greater power, at a cost of increasing the likelihood of obtaining type 1 errors, and suitable for exploratory research [18]. Cytokines were included in further analyses only when their amounts were significantly increased in the discovery cohort even after FDR correction.

Spearman's rank correlation was used to evaluate the association between cytokine levels and clinical variables (e.g., 6 MW distance, ABG, complete blood count, serum biomarkers) and the association of cytokine levels in serum and differential cell counts or cytokines in BALF.

Fisher’s exact test was used to evaluate the differences in the use of antifibrotic drugs between the two cohorts.

Survival time was calculated from the date of blood collection (baseline) until the patient’s death, with patients right-censored at the time of lung transplantation or time of last contact. Cox proportional hazard regression was used to evaluate the association of cytokine levels with overall survival, and the hazard ratios (HRs) were adjusted for age, a prognostic factor in a previous report of the IPF cohort in Japanese patients [19]. The association between cytokine levels and disease progression (a relative decline of 10% in FVC or 15% in DLCO or hospitalization) was also evaluated using the Cox proportional hazards model.

All statistical analyses were performed using JMP Pro 13.2.1, and a p-value < 0.05 was considered statistically significant.

## Results

### Patient characteristics

The characteristics of patients in the discovery and validation cohorts are summarized in Table 1. Patients in the discovery cohort were older, predominantly male, and more likely to be ever-smokers compared with controls. All patients in both cohorts were treatment-naïve at baseline, except for two in the discovery cohort who received pirfenidone therapy.

**Table 1:** Characteristics of study participants at baseline and outcomes

	Discovery cohort	Validation cohort	Control
Number	100	81	32
Age, years	69 (61, 75)*	72 (66, 79.5)*	61 (43, 67)
Male	86 (86)*	70 (86)*	18 (56)
Ever-smokers	91 (91)*	71 (88)*	8 (25)
%FVC	89.5 (73.7, 101.4)	79.5 (69.5, 89.2)	
%DLCO	42.7 (35.2, 53.9)	45.1 (33.4, 57.1)	
CPI	47.4 (37.3, 56.3)		
PaO_2_, Torr (n = 96)	82.7 (76.4, 89.1)	81.7 (73.8, 88.6)	
Six-minute walk distance, meter (n = 97)	463 (407, 520)	479.5 (421, 539)	
Lowest SpO_2_ during the six-minute walk test, % (n = 97)	88 (82, 92)	89 (83, 91)	
KL-6, IU/L	805 (607, 1260)	909 (595, 1600)	
SP-D, ng/mL	236.5 (132.3, 348)	206.5 (121.6, 339.3)	
LDH, IU/L	212 (193.8, 238.5)	223 (187.5, 249)	
CPFE	30 (30)		
Anti-fibrotic drug use at baseline	2 (2)	0 (0)	
Anti-fibrotic drug use more than 6 months during the observational period	51 (51)	47 (58)	
Mortality	52 (52)	19 (23)	
Observation period, months	41 (19, 68)	19 (12, 27)	

Data are expressed as medians (interquartile ranges) or numbers (percentages)

%FVC, the percentage of predicted forced vital capacity; %DLCO, the percentage of predicted diffusion capacity for carbon monoxide; CPI, composite physiologic index; PaO_2_, arterial pressure of oxygen; SpO_2_, percutaneous oxygen saturation; KL-6, Krebs von den Lungen-6; SP-D, surfactant protein-D; LDH, lactate dehydrogenase; CPFE, combined pulmonary fibrosis and emphysema

^*^P < 0.01 compared with controls (Mann–Whitney *U* test)

### Comparison of 48-plex measurements between the discovery cohort and the control group

**Fig. 1:**
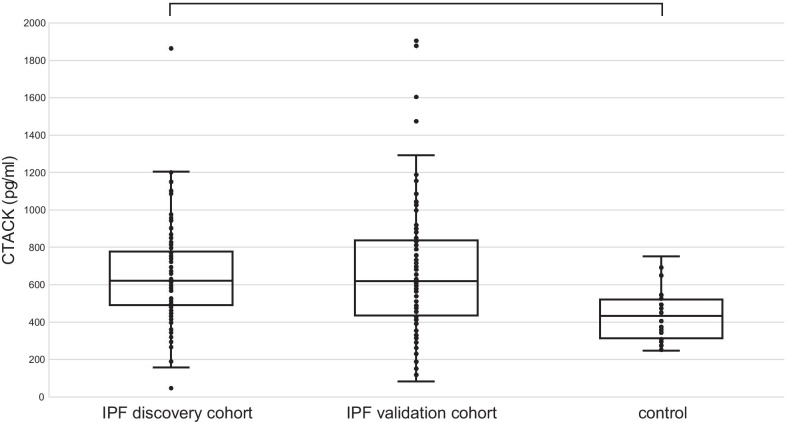
Serum CTACK levels in the discovery cohort (n = 100), validation cohort (n = 81), and controls (n = 32). Serum CTACK levels in the discovery [median (interquartile range), 620.9 (491.6–778.0) pg/mL] and the validation [619.5 (436.5–837.9) pg/mL] cohorts were higher than those in controls [434.1 (308.7–521.0) pg/mL]. The discovery cohort and the control group were compared using an analysis of covariance to adjust for age, sex, and smoking history, and the results were corrected by the False Discovery Rate (FDR) method. * *P* < 0.05

**Fig. 2:**
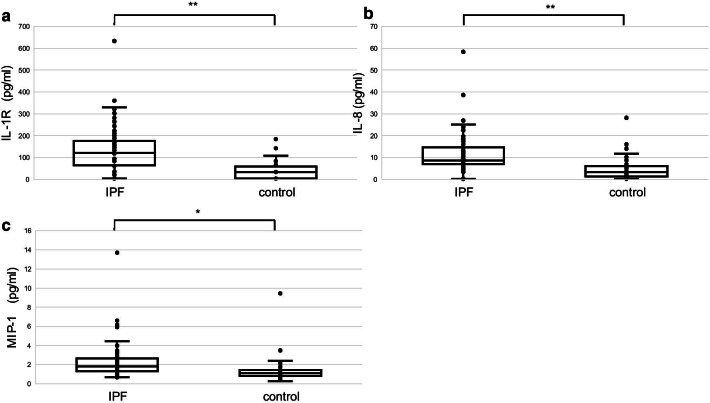
Comparison of the IL-1Rα, IL-8, and MIP-1α serum levels between IPF (n = 100) and controls (n = 32). Serum IL-1Rα [median (interquartile range): 121.9 (63.8–175.6) vs. 34.7 (4.4, 58.7) pg/mL, *P* < 0.01], IL-8 [8.7 (7.0–14.6) vs. 3.4 (1.3–6.1) pg/mL, *P* < 0.01], and MIP-1α [1.8 (1.3–2.7) vs. 1.1 (0.8–1.5) pg/mL, *P* < 0.05] levels were significantly higher in the discovery cohort than in controls. The discovery cohort and the control group were compared through analysis of covariance to adjust for age, sex, and smoking history, and the results were corrected by the FDR method. *P < 0.05, ** *P* < 0.01

Of the 48 multiplexed cytokines/chemokines, two (IL-1α and IL-12p40) were out of range (OOR) in ≥ 50% of cases in the discovery cohort, and 14 (IL-1α, IL-2, IL-3, IL-6, IL-10, IL-12p40, IL-12p70, IL-15, GM-CSF, GRO-α, LIF, MCP-3, β-NGF, and TNF-β) were OOR in ≥ 50% of cases in the control group. The remaining 34 cytokines/chemokines could be compared between the two groups (Additional file 1: Table S1). Among them, the expression of 18 cytokines (IL-1Rα, IL-2Rα, IL-4, IL-7, IL-8, IL-16, IL-17, Eotaxin, G-CSF, HGF, MCP-1, SCF, MIP-1α, PDGF-BB, RANTES, TNF-α, CTACK, and MIF) was significantly higher in the discovery cohort (IPF patients) than in the control group, even after adjustment for age, sex, and smoking history. A comparison of the serum levels of CTACK, IL-1Rα, IL-8, and MIP-1α between IPF patients and controls is illustrated in Figs. 1 and 2.

### Associations between serum cytokines/chemokines and outcome in the discovery cohort

During the median observation period of 41 months (IQR, 19–68), 51 patients (51%) were treated with antifibrotic drugs for > 6 months, 52 (52%) patients died, and 2 (2%) underwent lung transplantation in the discovery cohort.

Of the 18 cytokines/chemokines whose measurements were higher in the discovery cohort, four (IL-1Ra, IL-8, MIP-1α, and CTACK) were significantly associated with overall survival: IL-1Rα, HR = 1.04 (per 10 units), 95% CI 1.01–1.07; IL-8, HR = 1.04, 95% CI 1.01 − 1.08; MIP-1α, HR = 1.19, 95% CI 1.00 − 1.36; CTACK, HR = 1.12 (per 100 units), 95% CI 1.02 − 1.21 (Table 2).

**Table 2:** Univariable Cox proportional hazard models for the associations of serum cytokines and chemokines with overall survival in the discovery cohort

	Hazard ratio	95% CI	*P*-value
IL-1Rα, 10 units	1.04	1.01, 1.07	< 0.01
IL-8	1.04	1.01, 1.08	< 0.01
MIP-1α	1.19	1.00, 1.36	0.03
CTACK, 100 units	1.12	1.02, 1.21	0.01

CI, confidence interval; IL-1Rα, interleukin-1 receptor alpha; IL-8, interleukin-8; MIP-1α, macrophage inflammatory protein 1 alpha; CTACK, cutaneous T-cell-attracting chemokine

These four (IL-1Ra, IL-8, MIP-1α, and CTACK) were still significantly correlated with prognosis after adjusting for age and the use of antifibrotic drugs (IL-1Rα, HR = 1.04 [per 10 units], 95% CI 1.01 − 1.07, p-value < 0.01; IL-8, HR = 1.04, 95% CI 1.01–1.08, p-value < 0.01; MIP-1α, HR = 1.19, 95% CI 1.00– 1.36, p-value = 0.03; CTACK, HR = 1.12 (per 100 units), 95% CI 1.02–1.22, p-value = 0.01). None of these factors were associated with disease progression.

### Correlations of serum cytokines with clinical variables and BALF cytokines

The correlations of the serum levels of four cytokines (IL-1Ra, IL-8, MIP-1α, and CTACK) with various clinical variables (6MWT and serum biomarkers) are presented in Table 3. Of these four cytokines, only the level of IL-8 was significantly correlated between serum and BALF (Table 4). The correlation between serum levels of cytokines and BALF cell fractions were also examined. Neutrophil counts correlated with IL-8 and CTACK, and eosinophil counts correlated with IL-8 and MIP-α. No significant correlation was observed between cytokines and BALF total cell counts, lymphocyte counts, monocyte counts, or CD4/CD8 positive cell ratio (Table 5).

**Table 3:** Spearman's rank correlation coefficients for the associations of serum markers with physiologic parameters and serum biomarkers in the discovery cohort

	%FVC	%DLCO	CPI	6MWD	Lowest SpO_2_ at 6MWT	KL-6	LDH
IL-1Rα	− 0.30*	− 0.34*	0.32*	− 0.31*	− 0.15	0.24*	0.28*
IL-8	− 0.25*	− 0.24*	0.27*	− 0.30*	− 0.19	0.20*	0.21*
MIP-1α	− 0.18	− 0.17	0.12	− 0.20*	− 0.13	0.14	0.06
CTACK	− 0.12	− 0.25*	0.25*	− 0.27*	− 0.20*	− 0.02	− 0.00

Results with significant correlations (*P* < 0.05) are marked with *

6MWD, six-minute walk distance; 6MWT, six-minute walk test

**Table 4:** Spearman's rank correlation coefficients for the associations between serum and bronchoalveolar lavage fluid levels of IL-1Rα, IL-8, MIP-1α, and CTACK in the discovery cohort (n = 30)

	ρ	*P*-value
IL-1Rα	0.06	0.74
IL-8	0.39	0.03
MIP-1α	− 0.08	0.65
CTACK	0.01	0.96

ρ, Spearman's rank correlation coefficients

**Table 5:** Spearman's rank correlation coefficients for the correlation between differential cell counts and cytokines in bronchoalveolar lavage fluid levels of the discovery cohort (n = 30)

	TCC	Neut	Lym	Mono	Eo	CD4/8
IL-1Rα	0.20	0.32	− 0.17	− 0.08	0.36	− 0.08
IL-8	0.05	0.61*	− 0.13	− 0.24	0.54*	− 0.35
MIP-1α	0.28	0.33	0.03	− 0.23	0.45*	− 0.02
CTACK	0.09	0.48*	0.04	− 0.29	0.31	0.14

Results with significant correlations (*P* < 0.05) are marked with an *

TCC, total cell counts; Neut, neutrophil counts; Lym, lymphocyte counts; Mono, monocyte counts; Eo, eosinophil counts; CD4/8, CD4/CD8 ratio

### Associations between serum CTACK and outcome in the validation cohort

During the median observation period of 19 months (IQR, 12−27), 47 patients (58%) were treated with antifibrotic drugs for > 6 months. This frequency was not different from that in the discovery cohort (p-value = 0.37). During the follow-up period, 19 (23%) of the 81 patients died, and 1 (1%) underwent lung transplantation in the validation cohort.

Among the four cytokines associated with survival in the discovery cohort, CTACK was tested in the validation cohort because no pervious study had evaluated CTACK as a potential biomarker of disease progression and survival in IPF. Serum CTACK was again found to be significantly associated with survival in the validation cohort: HR = 1.14 (per 100 units), 95% CI 1.01–1.28; p-value = 0.03. CTACK was still significantly correlated with prognosis after adjusting for age and the use of antifibrotic drugs (HR = 1.15 [per 100 units], 95% CI 1.01–1.29, p-value = 0.02).

### Comparison of CTACK between CPFE and non-CPFE in the discovery cohort

In the discovery cohort, 30 (30%) of 100 patients were diagnosed with CPFE (Table 1). There was no significant difference in the value of CTACK between CPFE and non-CPFE (CPFE 679.1 [511.0–781.5], non-CPFE 613.7 [458.3–782.6], median [IQR], p-value = 0.38).

### Immunohistochemistry for CTACK and CCR10

**Fig. 3:**
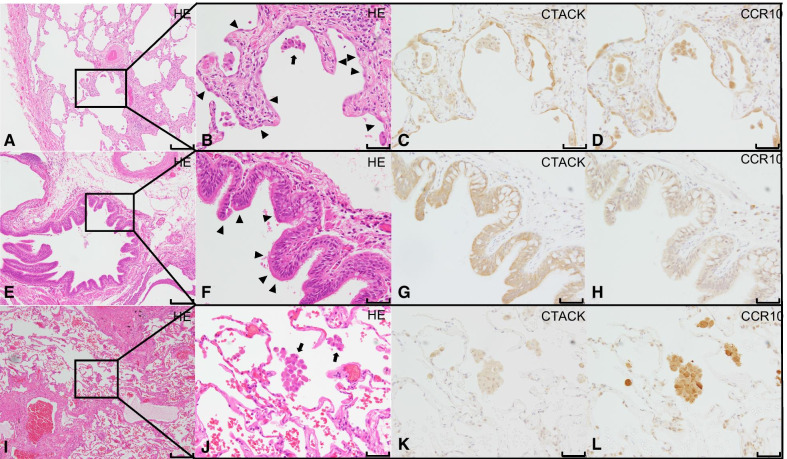
Immunohistochemical expression of CTACK and CCR10 and HE staining. The immunohistochemical expression of CTACK (**C**, **G**, **K**) and CCR-10 (**D**, **H**, **L**); hematoxylin and eosin (HE) staining (**A**, **B**, **E**, **F**, **I**, **J**) in idiopathic pulmonary fibrosis (IPF) (**A**–**D, E**–**H**) and control lung tissues (**I**–**L**). **A**, **E**, **I** are low power view and the black bar indicates 200 μm, **B**–**D**, **F**–**H**, **J**–**L** are high power view and the black bar indicates 50 μm. A is magnified in (**B**–**D**, **E**), **E** is magnified in (**F**–**H**, and **I**), and **I** is magnified in (**J**–**L**). Alveolar macrophages observed in the alveolar spaces, showing the positivity of both antibodies (arrows in **B**, **J**). Compared to the macrophages staining, alveolar epithelium were negative for the antibodies in the normal lung. In IPF lungs, CTACK and CCR-10 are expressed predominantly in type II alveolar epithelial cells (arrows heads in **B**) and peripheral bronchiolar epithelial cells (arrow heads in **F**). The CTACK and CCR-10 staining intensity in IPF lung tissue is stronger than that in control lung tissue

According to the results of the survival analysis in the discovery and validation cohorts, immunohistochemistry using anti-CTACK and anti-CCR10 antibodies was performed in lung specimens from IPF patients and controls. Both CTACK and CCR10 were significantly expressed in type II alveolar epithelial cells and in airway epithelial cells IPF lung tissue (Fig. 3).

## Discussion

In this study, we comprehensively measured levels of 48 cytokines/chemokines in sera from IPF patients and found that four cytokines/chemokines (IL-1Ra, IL-8, MIP-1α, and CTACK) were associated with prognosis. Among them, CTACK is a completely novel cytokine that has not been previously reported as a biomarker of IPF or interstitial lung diseases (ILDs). Therefore, we also measured serum CTACK levels in the validation cohort and confirmed its reproducibility as a prognostic marker in IPF. We also performed immunostaining in IPF lung and normal lung tissues for CTACK and found that CTACK was significantly more highly expressed in IPF lung tissue, primarily in type II alveolar epithelial cells and in airway epithelial cells. Furthermore, CCR10, a ligand of CTACK, was also expressed in a similar pattern as CTACK.

CTACK, also known as CCL27, was originally described as a CC chemokine receptor family member found in keratinocytes in psoriasis and other inflammatory and hyperproliferative skin conditions [20, 21]. CTACK participates in wound repair by recruiting T cells to the skin and inducing bone marrow-derived keratinocytes. In addition, in tumors, CTACK coordinates with VEGF to promote lymphangiogenesis; CTACK is also involved in tumor cell proliferation, migration, and angiogenesis [22, 23]. These findings suggest that in IPF, CTACK may contribute to fibrosis progression by inducing tissue inflammation, tissue repair, and angiogenesis.

As CTACK was previously believed to be a skin-specific chemokine, no study has investigated the role and localization of CTACK in the lungs. Bade et al. reported that the serum CTACK of COPD patients was higher than of healthy controls [24]. In the present study, we extracted CPFE cases based on HRCT images of IPF patients in the discovery cohort and compared the CTACK values of patients with and without emphysema; no significant difference was observed. This indicates that the present results were not influenced by the presence of emphysema. Based on multiplex measurements, we performed immunostaining to reveal the expression of CTACK and CCR10 (a ligand of CTACK) in lung specimens from IPF patients and controls. CCR10 and CTACK were expressed in similar patterns (primarily in type II alveolar epithelial cells and in airway epithelial cells) in IPF lung tissue, whereas expression was not observed in control lung tissue. Habiel et al. reported that CCR10, a ligand of CTACK, was expressed on lymphocytes (skin-directed T cells and IgA-producing B cells), plasmacytes, and alveolar epithelial cells in IPF patients [25]. They demonstrated that CCR10-positive epithelial cells were increased in IPF lungs and that transplantation of human-derived CCR10-positive epithelial cells into highly immunodeficient mice promoted lung remodeling. These results collectively suggest that serum CTACK may be of pulmonary origin and could be involved in lung remodeling.

On the contrary, no correlation was observed between CTACK levels in serum and BALF in our study. One possible explanation is that BAL is usually not performed in advanced honeycombed lungs because GGO and granular shadows that are considered areas of high inflammation are prioritized. Considering that staining of the lungs of IPF patients with anti-CTACK antibodies in this study also showed few stains in relatively normal areas, the effect of the site where BAL was performed may be a cause. Another possible explanation is that CTACK expressed on alveolar epithelial cells may move into the bloodstream through the basal membrane side, whereas a relatively small amount may transfer into the alveolar space. Accordingly, the CTACK level in BALF may not be a satisfactory biomarker of IPF.

In our study, three other cytokines/chemokines (IL-1Ra, IL-8, and MIP-1α) were also associated with IPF diagnosis and survival. Previous reports demonstrated the association of these three cytokines with the diagnosis and prognosis of IPF and other ILDs. As an antagonist of the IL-1 receptor, IL-1Ra inhibits IL-1 (both α and β) by binding to IL-1R on T cells and fibroblasts. When IL-1 and IL-1Ra levels are not balanced, lung diseases, such as IPF, progress [26, 27]. Smith et al. reported that tissue homogenates and BALF from patients with IPF both demonstrated elevated IL-1Ra expression compared with control subjects [28]. Barlo et al. reported that the IL-1Ra/IL-1β ratio in serum and BALF was significantly decreased in IPF patients compared with healthy controls. Korthagen et al. reported that lower levels of IL-1Ra predispose to the development of IPF and that genetic polymorphisms are involved in its development [27, 29]. These data collectively support that IL-1β is overexpressed relative to IL-1Ra in serum and BALF in IPF and that this imbalance may be involved in the pathogenesis of IPF. In this study, IL-1Ra and the IL-1Ra/IL-1β ratio (data not presented) were higher in IPF than in controls. The reasons for these conflicting findings are not known, but increased IL-1Ra expression might be a defensive reaction. IL-8 (CXCL8) is a neutrophil chemotactic factor. It has been reported that serum IL-8 levels were higher in IPF than in CTD-ILDs and that high serum IL-8 levels were correlated with poor prognosis in IPF [30, 31]. MIP-1α (CCL3) is a non-neutrophil inflammatory cell migration factor that was increased in BALF from IPF patients, and its serum level has been associated with the onset of ILD in systemic sclerosis [32, 33].

Serum and BALF biomarkers associated with disease progression or prognosis of IPF can be categorized into those that function in alveolar epithelial injury (e.g., KL-6 and MUC5B), extracellular matrix turnover (e.g., MMPs and periostin), and immunological changes (e.g., CCL18 and IL-6) [4, 34]. Some of these blood biomarkers are directly involved in the pathogenesis of IPF, and others appear as a result of lung damage. Cytokines and chemokines, such as CCL18 [35], IL-6 [36], CXCL13 [37], IL-8 (CXCL8) [38], and CTACK, may be directly involved in the pathogenesis of fibrosis and are thus potential targets for IPF treatment. Further investigation is necessary to determine the pathogenetic role and clinical significance of cytokines and chemokines in IPF.

In this study, the mortality rate of the discovery (52%) and validation (23%) cohorts was different. However, the difference in the mortality rate of the two cohorts was attributed to the different follow-up periods because there was no difference in the survival curves (data not shown). The mortality rate in the discovery cohort was comparable to that in the largest IPF cohort of Japanese subjects (mean survival duration: 35 months) [19].

Our study period from 2008 to 2019 was a period of significant change in the treatment of IPF with the advent of antifibrotic drugs. However, in Japan, pirfenidone has been available since 2008, and there was no significant difference in the use of antifibrotic drugs between the discovery (51%) and validation (58%) cohort. Moreover, the prognostic significance of the biomarker was demonstrated even after adjustment for the use of antifibrotic drugs. Therefore, the use of antifibrotic agents did not affect the study results.

In this study, four cytokines/chemokines associated with prognosis were not related to disease progression. This result was consistent with that reported previously wherein prognostic indicators were not necessarily indicators of disease progression in IPF [39].

This study has some limitations. First, both the discovery and validation cohorts were composed entirely of Japanese patients. Further studies involving multiracial cohorts are required. Second, this study did not clarify the pathogenetic role of CTACK in IPF. Third, the observation period in the validation cohort was not sufficiently long, although the serum CTACK level was significantly associated with survival.

## Conclusions

We demonstrated for the first time that CTACK is a potential serum biomarker of IPF and evaluated its localization in IPF lung tissue. By analyzing two independent cohorts and using immunohistochemistry, we concluded that CTACK may be a novel serum biomarker for predicting the prognosis of IPF. The roles of CTACK in the pathogenesis of IPF warrant further research.

## Supplementary Information

**Additional file 1: Table S1.** Results of 48-plex measurement in the sera of IPF patients (discovery cohort) and controls and the bronchoalveolar lavage fluid of IPF patients. Measurements (median and IQR) of 48 cytokines in the discovery cohort sera (n = 100) and the control group sera (n = 32), p-values for between-group comparisons, and the measurements of BALF in the discovery cohort (n = 30).

## Data Availability

The datasets used and/or analyzed during the current study are available from the corresponding author on reasonable request.

## Abbreviations

ABG: Arterial blood gas
BAL: Bronchoalveolar lavage
CCR10: CC chemokine receptor 10
CCL18: Chemokine CC motif ligand 18
CCL27: Chemokine CC motif ligand 27
CI: Confidence interval
CPFE: Combined pulmonary fibrosis and emphysema
CTACK: Cutaneous T-cell-attracting chemokine
HR: Hazard ratio
IPF: Idiopathic pulmonary fibrosis
IL-1Rα: Interleukin-1 receptor alpha
IL-8: Interleukin-8
IQR: Interquartile range
KL-6: Krebs von den Lungen-6
MIP-1α: Macrophage inflammatory protein 1 alpha
MMP: Matrix metalloproteinase
OOR: Out of range
6MWT: Six-minute walk test
SP-D: Surfactant protein-D
