# Supplementary material for: Cutaneous T-cell-attracting chemokine as a novel biomarker for predicting prognosis of idiopathic pulmonary fibrosis: a prospective observational study
Source: Respir Res. 2021 Jun 17;22:181. doi: 10.1186/s12931-021-01779-9 (PMC8218397; doi:10.1186/s12931-021-01779-9)
Supplement: Supplementary file 1 — Additional file 1: Table S1. Results of 48-plex measurement in the sera of IPF patients (discovery cohort) and controls and the bronchoalveolar lavage fluid of IPF patients. Measurements (median and IQR) of 48 cytokines in the discovery cohort sera (n = 100) and the control group sera (n = 32), p-values for between-group comparisons, and the measurements of BALF in the discovery cohort (n = 30). [file 12931_2021_1779_MOESM1_ESM.docx]

Additional Table S1. Results of 48-plex measurement in the sera of IPF patients (discovery cohort) and controls and the bronchoalveolar lavage fluid of IPF patients

| (pg/mL) | IPF, serum  (discovery cohort, n = 100) | |  | Control, serum  (n = 32) | |  | Adjusted  *P*-value* |  | IPF, BAL  (n = 30) | |
| --- | --- | --- | --- | --- | --- | --- | --- | --- | --- | --- |
| IL-1α | - | - |  | - | - |  |  |  | 8.6 | (6.3, 12.3) |
| IL-1Rα | 121.9 | (63.8, 175.6) |  | 34.7 | (4.4, 58.7) |  | <0.01 |  | 540.4 | (322.1, 699.9) |
| IL-1β | 0.4 | (0.2, 0.9) |  | 0.3 | (0.2, 0.7) |  | 0.47 |  | 1.1 | (0.8, 1.4) |
| IL-2 | 0.3 | (0.3, 2.6) |  | - | - |  | - |  | 0.5 | (0.2, 1.5) |
| IL-2Rα | 40.8 | (29.1, 59.6) |  | 22.2 | (17.0, 27.9) |  | 0.03 |  | 5.6 | (4.3, 6.7) |
| IL-3 | 0.02 | (0.02, 0.17) |  | - | - |  | - |  | 2.1 | (1.8, 2.8) |
| IL-4 | 1.5 | (1.1, 2.2) |  | 0.8 | (0.3, 1.4) |  | <0.01 |  | 0.6 | (0.5, 0.7) |
| IL-5 | 33.0 | (14.4, 47.1) |  | 12.3 | (1.4, 26.0) |  | 0.24 |  | 20.6 | (18.3, 23.4) |
| IL-6 | 0.9 | (0.3, 1.6) |  | - | - |  | - |  | 2.3 | (1.8, 4.2) |
| IL-7 | 17.2 | (13.7, 22.6) |  | 7.3 | (4.2, 9.8) |  | <0.01 |  | 5.3 | (4.3, 6.3) |
| IL-8 | 8.7 | (7.0, 14.6) |  | 3.4 | (1.3, 6.1) |  | <0.01 |  | 58.1 | (34.2, 106.0) |
| IL-9 | 40.6 | (30.9, 53.4) |  | 32.4 | (21.4, 107.3) |  | 0.085 |  | 10.1 | (9.2, 12.9) |
| IL-10 | 1.0 | (0.2, 2.1) |  | - | - |  | - |  | 4.4 | (4.0, 4.8) |
| IL-12p40 | - | - |  | - | - |  | - |  | - | - |
| IL-12p70 | 0.8 | (0.5, 4.0) |  | - | - |  | - |  | 0.6 | (0.2, 0.9) |
| IL-13 | 4.5 | (3.7, 6.9) |  | 2.5 | (0.2, 3.8) |  | 0.074 |  | 1.2 | (1.1, 1.3) |
| IL-15 | 31.6 | (31.6, 159.8) |  | - | - |  | - |  | 164.8 | (155.0, 176.0) |
| IL-16 | 32.8 | (23.0, 47.0) |  | 16.1 | (11.0, 21.0) |  | 0.030 |  | 28.2 | (18.0, 49.4) |
| IL-17 | 11.4 | (8.8, 16.5) |  | 5.0 | (2.5, 6.8) |  | <0.01 |  | 3.3 | (2.9, 4.0) |
| IL-18 | 25.1 | (16.6, 39.3) |  | 14.8 | (11.2, 22.3) |  | 0.23 |  | 7.7 | (5.9, 15.1) |
| Eotaxin | 52.3 | (40.8, 67.0) |  | 22.5 | (18.9, 28.1) |  | <0.01 |  | 1.6 | (1.3, 2.9) |
| FGF basic | 16.8 | (9.1, 22.6) |  | 15.0 | (7.4, 22.2) |  | 0.50 |  | 7.8 | (5.9, 9.1) |
| G-CSF | 81.1 | (59.5, 110.1) |  | 23.3 | (16.9, 37.1) |  | <0.01 |  | 44.7 | (32.7, 77.6) |
| GM-CSF | 2.4 | (0.5, 4.8) |  | - | - |  | - |  | 1.4 | (1.2, 2.0) |
| IFN-γ | 2.3 | (1.7, 3.1) |  | 1.5 | (0.9, 3.7) |  | 0.27 |  | 11.5 | (9.4, 17.0) |
| GROα | 150.1 | (105.7, 186.8) |  | - | - |  | - |  | 1260.9 | (851.1, 1597.8) |
| HGF | 231.0 | (162.0, 281.1) |  | 111.7 | (80.5, 167.2) |  | <0.01 |  | 36.3 | (29.4, 49.4) |
| IFN-α2 | 6.0 | (2.5, 10.3) |  | 6.1 | (2.5, 8.6) |  | 0.71 |  | 6.3 | (4.4, 7.6) |
| LIF | 0.07 | (0.07, 5.78) |  | - | - |  | - |  | 20.2 | (16.6, 23.8) |
| MCP-3 | 0.3 | (0.3, 0.9) |  | - | - |  | - |  | 3.1 | (1.5, 5.8) |
| IP-10 | 294.7 | (216.5, 414.3) |  | 190.9 | (157.3, 307.9) |  | 0.21 |  | 247.0 | (146.3, 422.7) |
| MCP-1 | 30.2 | (19.1, 12.4) |  | 9.0 | (6.5, 12.4) |  | <0.01 |  | 107.3 | (42.1, 304.0) |
| MIG | 158.5 | (108.7, 244.5) |  | 75.8 | (54.2, 91.7) |  | 0.24 |  | 124.1 | (73.1, 323.6) |
| β-NGF | 0.05 | (0.04, 0.63) |  | - | - |  | - |  | 0.90 | (0.7, 1.1) |
| SCF | 76.5 | (55.1, 102.0) |  | 45.4 | (23.6, 54.0) |  | 0.013 |  | 12.6 | (10.3, 14.8) |
| SCGF-BB | 105274.1 | (85280.5, 124742.1) |  | 89713.3 | (82403.7, 104014.9) |  | 0.081 |  | 1639.1 | (1273.5, 1962.9) |
| SDF-1α | 138.6 | (115.4, 165.7) |  | 121.6 | (104.3, 164.3) |  | 0.11 |  | 12.6 | (9.9, 15.8) |
| MIP-1α | 1.8 | (1.3, 2.7) |  | 1.1 | (0.8, 1.5) |  | 0.012 |  | 1.2 | (0.7, 4.1) |
| MIP-1β | 29.8 | (23.8, 39.3) |  | 17.9 | (14.6, 83.7) |  | 0.056 |  | 10.1 | (5.1, 20.9) |
| PDGF-BB | 1000.7 | (570.8, 1479.7) |  | 130.5 | (63.9, 671.0) |  | <0.01 |  | 17.0 | (13.4, 19.7) |
| RANTES | 6316.5 | (4631.1, 7496.4) |  | 1331.5 | (918.0, 4602.4) |  | <0.01 |  | 15.9 | (10.4, 43.5) |
| TNF-α | 11.7 | (10.0, 14.7) |  | 7.8 | (3.4, 12.0) |  | <0.01 |  | 9.9 | (8.4, 11.9) |
| VEGF | 126.6 | (52.7, 1440.6) |  | 181.7 | (7.3, 2660.0) |  | 0.69 |  | 88.4 | (74.7, 105.8) |
| CTACK | 620.9 | (491.6, 778.0) |  | 434.1 | (308.7, 521.0) |  | 0.043 |  | 2.8 | (1.9, 3.8) |
| MIF | 460.0 | (356.8, 630.1) |  | 202.8 | (156.8, 285.9) |  | <0.01 |  | 2791.7 | (2135.6, 4116.5) |
| TRAIL | 38.1 | (30.8, 46.7) |  | 43.1 | (29.6, 57.1) |  | 0.44 |  | 18.7 | (13.5, 28.3) |
| M-CSF | 9.7 | (7.2, 12.2) |  | 7.7 | (5.9, 10.7) |  | 0.87 |  | 10.6 | (7.5, 20.5) |
| TNF-β | 0.01 | (0.01, 0.46) |  | - | - |  | - |  | 6.2 | (5.2, 8.3) |

Data are expressed as medians (interquartile ranges).

*Serum values are compared between IPF patients (discovery cohort) and controls. Using FDR methods, *P*-values were calculated after adjustments for age, sex, and smoking history. Results were considered significant if *P* < 0.05.

Abbreviations: IPF, idiopathic pulmonary fibrosis; BAL, bronchoalveolar lavage
